# Supplementary material for: Macromonas nakdongensis sp. nov., Isolated from Freshwater and Characterization of Bacteriophage BK-30P—The First Phage That Infects Genus Macromonas
Source: Microorganisms. 2023 Sep 5;11(9):2237. doi: 10.3390/microorganisms11092237 (PMC10535371; doi:10.3390/microorganisms11092237)
Supplement: Supplementary file 1 [file microorganisms-11-02237-s001.zip › microorganisms-2577955-supplementary.pdf]

Supplementary Materials

Macromonas nakdongensis sp. nov., Isolated from Freshwater and Characterization of Bacteriophage BK-30P—the First Phage That Infects Genus *Macromonas*

Kiwoon Baek[1,2], and Ahyoung Choi[2,*]

[1]Department of Biological Sciences and Bioengineering, Inha University, Incheon 22212, Republic of Korea.

[2]Nakdonggang National Institute of Biological Resources, 137 Donam 2-gil, Sangju 37242, Republic of Korea

[*] Correspondence: aychoi@nnibr.re.kr; Tel.: +82-54-530-0891; Fax: +82-54-530-0899

Figure S1. Transmission electron micrograph of BK-30^T cells. Bar, 0.2 μm.

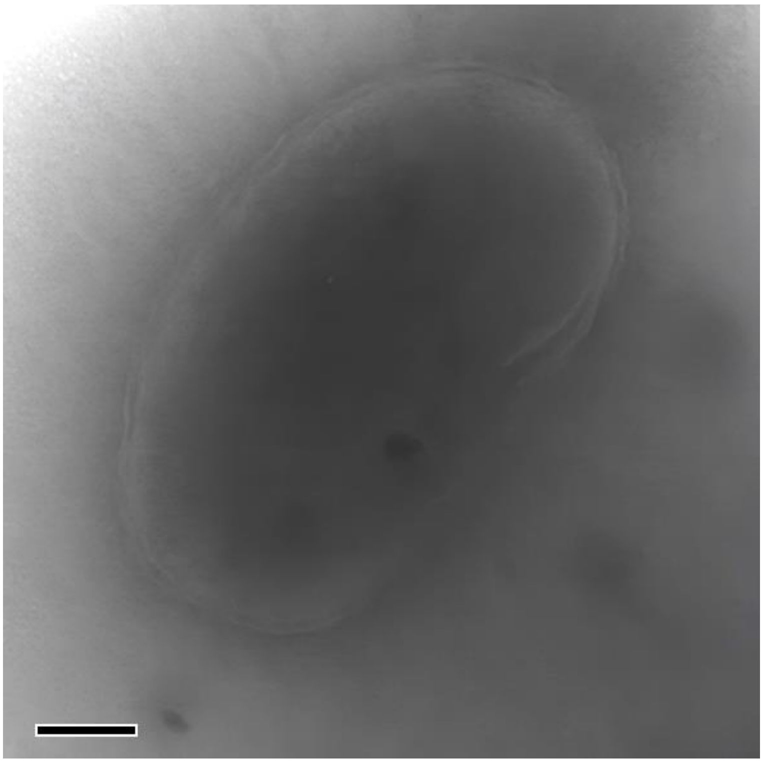

Figure S2. Subsystem category distribution of strain BK-30^T based on the RAST annotation server (https://rast.nmpdr.org/) (accessed on 24 August 2023).

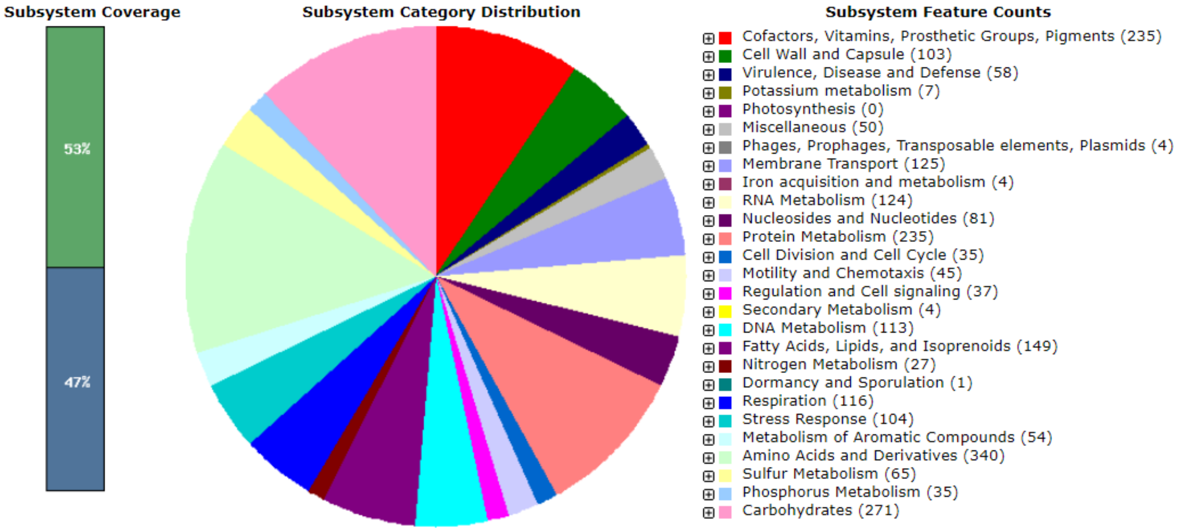

Figure S3. Genome map of Macromonasphage BK-30P. The total length of the genome is 43,064 bp. The arrow colors indicate putative functional categories (Green: structure; Blue: DNA packaging; Red: DNA replication and metabolism; Gray: hypothetical proteins).

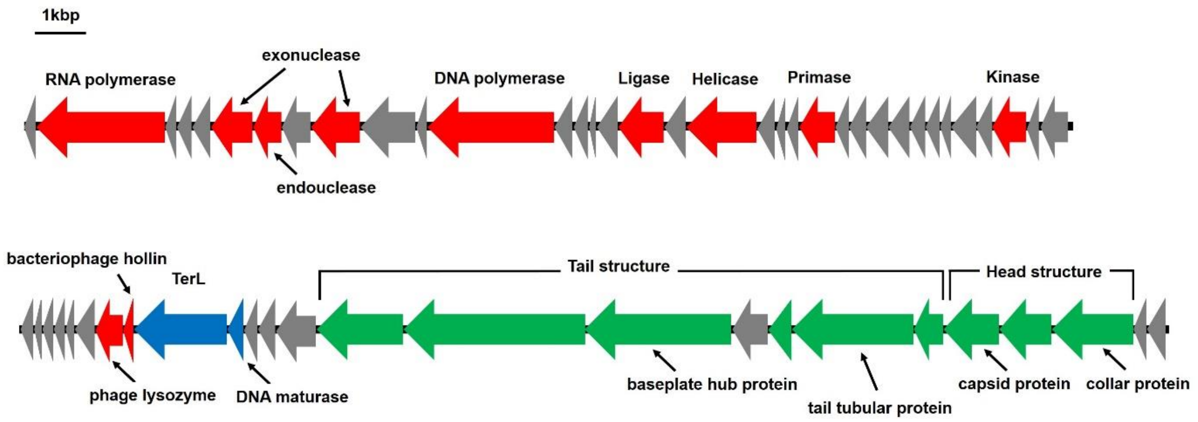

Figure S4. The relationship between Macromonasphage BK-30P (shown in bold) and other phages is depicted in a phylogenetic tree. The tree is built on aligned *TerL* sequences, with the Bacillus phage SPO1 serving as an outgroup. Except for BK-30P, all sequences were obtained from NCBI and aligned using CLUSTALW before their evolutionary relationships were inferred using the maximum likelihood method in MEGA6. The bootstrap consensus was set at 1000 replicates, and branch nodes displayed bootstrap values (>60%). Bar, 0.2 substitutions per amino acid position.

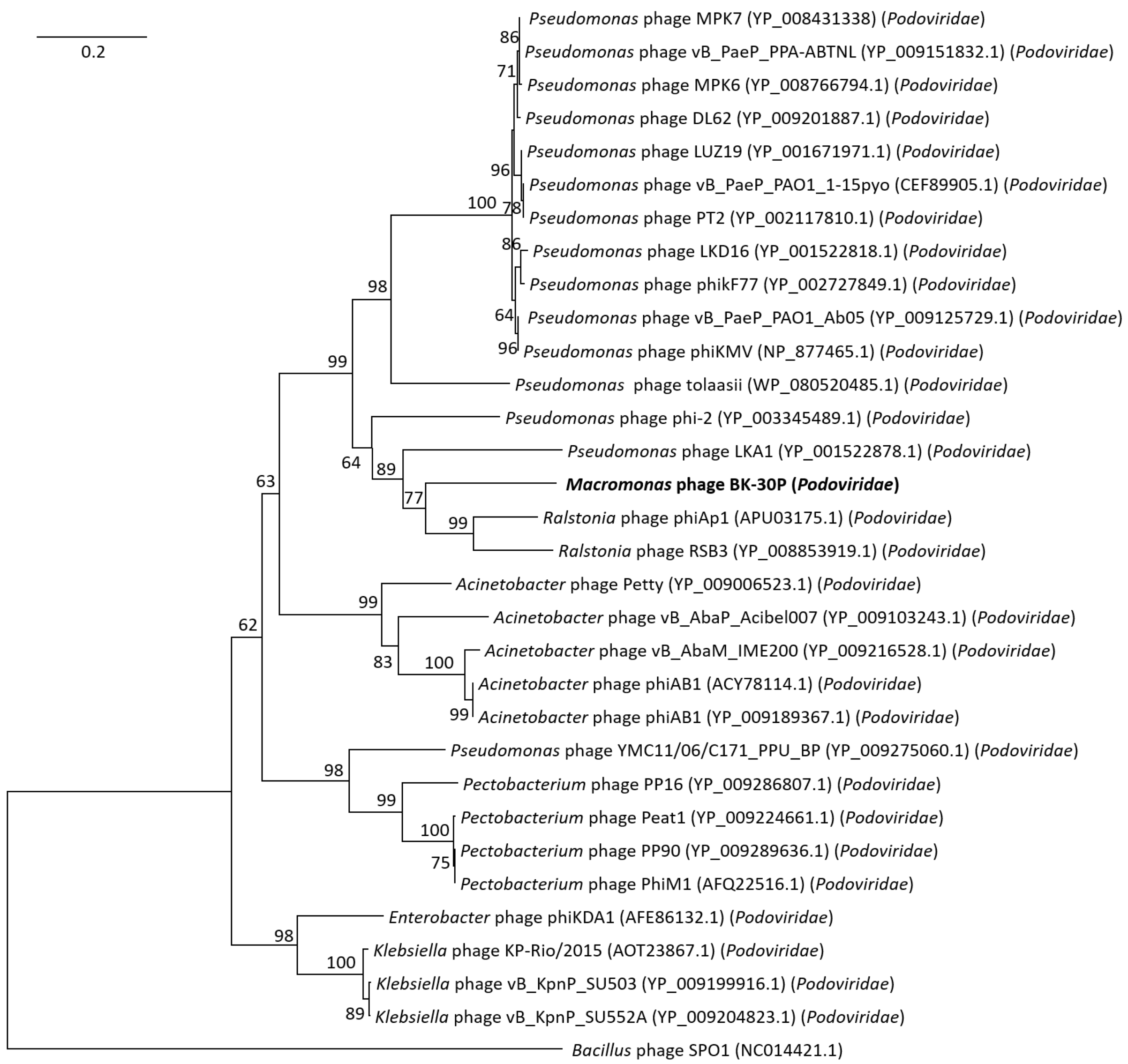

Table S1. Summary of the draft genomes of strain BK-30^T and *Macromonas bipunctata* DSM 12705^T. Draft genomes for strain BK-30^T and *Macromonas bipunctata* DSM 12705^T are available at the NCBI whole genome shotgun sequencing project (WGS) under the accession numbers NWMV00000000 and NWBQ00000000, respectively.

**Features**	***M. nakdongensis* BK-30^T**	***M. bipunctata* DSM 12705^T**
Sequencing platform	Illumina MiSeq	Illumina MiSeq
Assembler	CLC Workbench v9.0	CLC Workbench v9.0
Genome size (bp)	3,064,983	2,699,505
GC content (%)	67.3	63.8
Coding sequence (CDS)	2,837	2,457
rRNA genes (5S, 16S, 23S)	3 (1, 1, 1)	3 (1, 1, 1)
tRNA genes	53	49
No. of Contigs	182	115

Table S2. Genome statistics of Macromonasphage BK-30P.

Attribute	Value	% of total
Genome size (bp)	43,064	100.0
DNA coding (bp)	41,525	95.8
DNG G+C (bp)	25,235	51.0
Total genes	60	100.0
Protein coding genes	60	100.0
RNA genes	0	0.0
Genes with function prediction	21	25.0
Genes assigned to COGs	8	15.4
Genes with Pfam domains	13	25.0
Genes with signal peptides	0	0.0
Genes with transmembrane helices	4	7.7

Table S3. Functional annotation of the ORFs predicted in the Macromonasphage BK-30P genome.

ORF	Strand	Predicted function	Most significant hit in BLASTP against nr (organism, GenBank accession no., E-value)[1]	Domain or Family (function, e-value)[2]
1	-	hypothetical protein	-	-
2	-	RNA polymerase	phage-specific RNA polymerase (Pseudomonas phage LUZ19, YP_001671971.1, 1E-170)	PF00940 (RNA polymerase, 4E-94)/ COG5108 (DNA-directed RNA polymerase, 2E-73)
3	-	hypothetical protein	-	-
4	-	hypothetical protein	hypothetical protein (Janthinobacterium lividum, WP_010396924.1, 2E-25)	-
5	-	hypothetical protein	p33 (Xanthomonas phage Xop411, YP_001285703.1, 5E-34)	-
6	-	exonuclease	Exonuclease (Pseudomonas phage DL62, YP_001522875.1, 1E-89)	PF13482 (RNase_H_2, 1.7E-6)
7	-	endonuclease	DNA endonuclease VII (Pectobacterium phage PP16, YP_009286799.1, 4E-49)	PF02945 (Recombination endonuclease VII, 6.4E-14)
8	-	hypothetical protein	-	-
9	-	exonuclease	Exonuclease (Ralstonia phage RSB3, YP_008853914.1, 5E-96)	-
10	-	hypothetical protein	structural protein (Pectobacterium phage PPWS1, BAS69536.1, 7E-56)	-

11	-	hypothetical protein	-	-
12	-	DNA polymerase	DNA polymerase (Cronobacter phage vB_CskP_GAP227, YP_007348339.1, 4E-175)	PF00476 (DNA polymerase, 1.9E-30)/ COG0749(polymerase domains, 3E-16)
13	-	hypothetical protein	-	-
14	-	hypothetical protein	-	-
15	-	hypothetical protein	-	-
16	-	hypothetical protein	hypothetical protein (Aureimonas sp. AU40, WP_062120293.1, 2E-48)	-
17	-	DNA ligase	DNA ligase (Ralstonia virus phiAp1, APU03163.1, 5E-80)	PF01068 (DNA ligase, 1E-10)/ COG1793(ATP-dependent DNA ligase, 2E-06)
18	-	hypothetical phage protein	Bacteriophage hypothetical protein (Paraburkholderia xenovorans LB400, ABE29876.1, 2E-55)	-
19	-	DNA helicase	DNA helicase (Pseudomonas phage LKA1, YP_001522864.1, 2E-159)	PF13481.4 (AAA domain, 2.7E-09)
20	-	hypothetical protein	-	-
21	-	hypothetical protein	-	-
22	-	hypothetical protein	-	-
23	-	DNA primase	DNA primase (Ralstonia phage RSB3, YP_008853904.1, 6E-37)	PF13155 (Toprim_2, 4.1E-06)

24	-	hypothetical protein	-	-
25	-	hypothetical protein	-	-
26	-	hypothetical protein	hypothetical protein (Ralstonia phage RS-PII-1, APU00321.1, 5E-04)	-
27	-	hypothetical protein	-	-
28	-	hypothetical protein	-	-
29	-	hypothetical protein	-	-
30	-	hypothetical protein	-	-
31	-	hypothetical protein	-	-
32	-	hypothetical protein	-	-
33	-	Protein kinase	protein kinase (Serratia phage SM9-3Y, AOZ61236.1, 7E-05)	-
34	-	hypothetical protein	-	-
35	-	hypothetical phage protein	hypothetical protein vBAbaPPDAB9_48 (Acinetobacter phage vB_AbaP_PD-AB9, YP_009189871.1, 2E-52)	-
36	-	hypothetical protein	-	-
37	-	hypothetical protein	-	-
38	-	hypothetical protein	-	-

39	-	hypothetical protein	-	-
40	-	hypothetical protein	-	-
41	-	hypothetical protein	-	-
42	-	Phage lysozyme	lysozyme (Pseudomonas, WP_054897643.1, 7E-45)	PF00959 (Phage lysozyme, 2.9E-21)/ COG3772 (Phage-related lysozyme, 3E-25)
43	-	Bacteriophage holin	prophage Hp1 family holin (Yersinia frederiksenii, CNK75783.1, 3E-13)	PF16080 (Bacteriophage holin family HP1, 6.3E-16)
44	-	terminase large subunit	terminase large subunit (Pseudomonas phage phikF77, YP_002727866.1, 2E-179)	COG5410(Uncharacterized protein, 9E-78)
45	-	DNA maturase	DNA maturase A ([Ralstonia phage RSB3, YP_008853933.1, 5E-15]	-
46	-	hypothetical protein	-	-
47	-	hypothetical protein	hypothetical protein SAMN03159494_03594 (Achromobacter sp. NFACC18-2, SEJ85374.1, 2E-25)	-
48	-	hypothetical protein	hypothetical protein (Burkholderia ubonensis, WP_059925242.1, 5E-24)	-
49	-	tail fiber protein	tail fiber protein (Ralstonia phage RSB3, YP_008853931.1, 4E-32)	PF03906 (Phage T7 tail fiber protein, 2.8E-14)
50	-	hypothetical phage protein	internal virion protein (Ralstonia virus phiAp1, APU03187.1, 2E-168)	-

51	-	Phage baseplate hub	internal virion protein (Ralstonia virus phiAp1, APU03186.1, 2E-82)	-
52	-	hypothetical protein	-	-
53	-	tail fiber protein	tail fiber protein (Ralstonia phage RSK1, YP_008853798.1, 2E-27)	COG1917 (Cupin domain protein related to quercetin dioxygenase, 3E-22)
54	-	tail tubular protein	tail tubular protein B (Vibrio phage VP93, YP_002875655.1, 2E-156]	-
55	-	tail tubular protein	tail tubular A (Ralstonia virus phiAp1, APU03182.1, 1E-50)	PF17212 (Tail tubular protein, 1.2E-21)
56	-	Phage capsid protein	capsid protein (Ralstonia phage RSB3, YP_008853924.1, 6E-149)	-
57	-	scaffolding protein	scaffolding protein (Ralstonia phage RSB3, YP_008853923.1, 5E-32)	-
58	-	Phage collar	head-tail connector protein (Ralstonia phage RSB3, YP_008853922.1, 1E-152)	PF12236 (Bacteriophage head to tail connecting protein, 1.5E-42)
59	-	hypothetical protein	-	-
60	-	hypothetical phage protein	hypothetical protein AV952_gp40 (Pseudomonas phage LKA1, YP_001522880.1, 6E-15)	PF00583 (Acetyltransferase family, 6.1E-06)

[1] Best BLASTP match from NCBI nr database (e-value ≤ 10^{-4}).

[2] From the search results of COG, Pfam, and TIGRFAM, the one with the most significant e-value (≤10^{-4}) was presented.
